# American Society of Dental Science

**Published:** 1895-05

**Authors:** 

**Affiliations:** American Academy of Dental Science


### Reports of Society Meetings

---

### AMERICAN ACADEMY OF DENTAL SCIENCE.

The regular monthly meeting of the American Academy of Dental Science was held at Young's Hotel, Boston, Wednesday, October 3, at six o'clock, President Smith in the chair.

The paper for the evening was read by Robert W. Greenleaf, A.M., M.D., professor of Materia Medica, Massachusetts College of Pharmacy. Subject, "The Relation of Modern Therapeutics to the Practice of Dentistry."

(For Professor Greenleaf's paper, see page 152.)

### DISCUSSION.

*President Smith.*—We have before us a very interesting subject, which has been very ably presented. According to the programme the discussion will be opened by Dr. E. C. Briggs, assistant professor of Materia Medica in the Dental Department of Harvard University.

*Dr. Briggs.*—I think I voice the sense of the meeting when I say that this is a most interesting and sound paper of Professor Greenleaf's. It might be said that he has represented the question and discussed it himself pretty thoroughly.

Such a paper as this cannot be otherwise than a spur to the older practitioners and an encouragement to the younger ones, who, I trust, are coming into the field with more of the foundation principles of therapeutics than was formerly the case. Only within recent years have I been able to clear from the catalogue of our school the term "dental materia medica;" as if there could be such a thing as "*dental* materia medica." The materia medica taught in the dental school at Harvard to-day is the materia medica of the whole field, and, according to the announcement of other colleges, that standard is being approached more and more all the time. Formerly the student was taught the use of certain drugs for particular conditions which he was brought in contact with.

I think we advanced long ago beyond the "make-believe" period, but we landed in the empiric stage, and there we have remained.

Somebody announces that he has used a certain thing for a certain purpose and it has accomplished the result desired, and immediately all begin to use it; and then somebody comes out and announces another thing that he is using, and like sheep all flock to that. In the Harvard Dental School the *principles* that underlie the action of all drugs are taught, and the aim is that the student shall understand what the principle is for which he uses a certain drug. And if he is not obtaining in the best manner the result desired, then he is ready to resort to some other drug if it contains the same active principle. We are getting down more and more to the principles of things and are abandoning the use of things either in the "make-believe" or the "empiric" form. It has been a little hard for us to get to that position, because so many of us have tried hard to maintain that the dental profession was a separate profession, and what we did, we did from a dental point of view; and we had a dental materia medica, dental surgery,—everything dental.

We are gradually finding out, however, that the best advancement even in the dental sense is made through the lines of modern therapeutics. I do not mean by that through the increased use of drugs, but modern therapeutics has given us drugs for the production of antisepsis and anæsthesia which are of invaluable service. The knowledge of the principles of these various drugs belongs not to the therapeutist, but to medicine, and this knowledge brings us to the point that we cannot help being specialists in medicine.

In the case of our Miller, who has done so much for the entire field of medicine, a great deal of the benefit to us has come more quickly because he was a dentist, and we follow his recommendations as if they pertained strictly to the dentist, whereas his researches, as we know, are applicable to all branches of medical science.

I shall not go into the details of the application of these drugs; the discussion will occupy enough of the evening. It is sufficient to say that the advice of this paper and the tendency of the times is to struggle more and more towards a thorough understanding of the principles and not following the empiric form of treating cases and applying drugs. Because such and such a thing has been advised and prescribed by some one who claims to have had good success with it, is not a sufficient justification for using it; we must know the effect we wish to obtain and the active principles of the drug which we are using to obtain it.

*President Smith.*—The subject is now open for general discussion.

To the guests present I would extend, in the name of the Academy, the privileges of the floor.

*Dr. Williams.*—I wish to express my appreciation of the scientific presentation of the subject which has been treated by Professor Greenleaf. The remarks in the first part of his paper remind me of the advice given by Dr. Weir Mitchell before a society of ophthalmologists. He drew their attention to the tendency in that specialty of limiting their thoughts to their specialty, so that their minds were not able to comprehend the relations existing between the eye and other parts of the body, and he showed them that the effect of such a course was a deficiency of knowledge which rendered them unable to practise to advantage even their own specialty.

Instruction in the principles of a science is a matter that I have for years advocated; and I have had several talks with President Eliot and others in regard to the importance of the fundamental principles in which this specialty as well as others should be educated.

I have sometimes illustrated the effect of a man confining his thoughts in one direction in this way: Suppose an accountant educated only within the limits of the addition, subtraction, or multiplication tables. Everything that comes along within the limits of those tables is promptly and accurately done; he does his accounting by rote, as it were. Give him something outside of the tables requiring knowledge of the more intricate principles of arithmetic, and he is unable to solve the sum. There has been too much of such practice in the dental school's instruction. The student is told, "This will do so and so," but no reason is assigned why it will do so, reasons which would be derived from the knowledge of *principles.* I also illustrated this matter some time ago in this way: Take the science of agriculture, which covers so broad a field that it is impossible for one man to understand all its branches; and it is necessarily divided into departments, such as the cultivation of fruits, flowers, and vegetables. It may be that one man's specialty is the cultivation of flowers; another understands the production of fruit and vegetables for the market, and so on, each man having a special knowledge of some one branch. But each must understand the *general principles* of agriculture as relating to any one of those specialties, as, for example, the effects of climate, the different kinds of soil in which the various plants will thrive best. And no matter how fine a knowledge a man may possess of any of the branches, if he is ignorant of those general principles, he is likely to make failures.

I think that when Professor Greenleaf's paper appears in the

International Dental Journal it will be a great encouragement in the matter of general education.

*Dr. Bradley.*—I am somewhat interested in this subject, as we all are. Very many times in our profession we feel that some systemic remedy would be of assistance to our patients in inflamed conditions of the oral cavity. I very frequently inquire as to the systemic condition of the patient when there are unusually inflamed conditions,—that is, as to whether they are troubled with costiveness, biliousness, or anything of that nature, and suggest the use of slight cathartic medicines, and in the more marked cases consultation with their physician. In persistent cases of Rigg's disease I nearly always ask my patients if they have a gouty diathesis or any difficulty with the kidneys, and suggest a consultation with the physician. In some cases, where the patient has taken my advice, I have been very much disappointed to have the doctor tell them, "You have nothing of the kind," without ever making what it would seem to me a proper investigation of the matter, such as an analysis of the urine or a proper inquiry into the condition of the patient; and I have wondered if there was not some way in which such things could be ventilated and discussed, so that the two professions could work harmoniously together. The question has occurred to me whether it would be advisable that we should take up a course of study in dentistry which would include the analysis of urine,—I think such special instruction is given in some of the schools,—and we would then be more positive as to the best thing to be done for the patient. I merely mention those things in the hope that it will bring forth a discussion as to whether it would be advisable to send patients to a physician to have an examination made to detect any systemic difficulty, and whether we feel they would make a proper examination, or whether it would be advisable to sacrifice a little of our time for the purpose of equipping ourselves with a knowledge sufficient to make the examination.

*President Smith.*—I would simply say, in answer to Dr. Bradley, that the examination of the urine, which is included in the instruction under the head of medical chemistry, is taught in the Dental Department of Harvard University.

*Dr. Bradley.*—Thank you; I am glad to hear that it is. Of course, the matter of prescribing for such affections as I have mentioned comes entirely outside of our province. I do not presume to say that the dentist should take it on himself to give prescriptions in such cases, because in many instances the difficulty requires the assistance of physician and dentist, and we two should go hand in hand in the treatment of disease.

*President Smith.*—One of our members has made investigations in therapeutics in a different school from the one which some of us adopt, and claims success. I do not know whether to present him to you to-night as from Chicago, or Cambridge, or Boston, but I know you would all like to hear from Dr. Taft.

*Dr. C. H. Taft.*—I want to add my word of appreciation of Dr. Greenleaf's paper. I must confess that in my experience and practice of dentistry within the last eight years, and more especially within the last three or four years, since I have begun to bring the application of therapeutics and a better knowledge of the materia medica into my practice for the treatment of cases, where I was wholly at sea with such kind of local treatment as I had been taught in the school, that I have questioned very seriously whether the dentist who makes a special study of therapeutics, and gives the results of such study subsequently to the profession, receives the support which it would seem that he should receive. I have been working in the field of therapeutics for some time past, putting a great deal of study for the benefit of my patients and for the purpose of giving to the profession the results of my observations and investigations, and I must frankly say that I do not think I have at all times received the encouragement from the profession to continue such study that I naturally feel I ought to have had. However, that counts for but very little, for I know that my patients have received the benefit of my work. I was glad to hear Dr. Briggs take the ground which he did in opening the discussion of Professor Greenleaf's paper, for it calls to mind the fact that about a year ago he read a paper before the Harvard Odontological Society, under the title of "The Dentist as a Prescriber of Drugs." I was in practice in Chicago at the time, and I remember with what interest I read the paper and the discussion upon it. Dr. Briggs took the ground that I was leading the profession into rather dangerous territory; that I was drawing them out of their legitimate field of work in encouraging the prescribing of drugs systemically for the treatment of any lesion that we might be called upon as specialists to treat where all other efforts had failed. But I am glad to see to-night that he has taken a little more liberal ground. If we are to give any special attention to the materia medica as it is taught in the dental schools, we must ask ourselves the question, What is the use of *teaching* the materia medica, if it is not to give the student a good general knowledge of drugs, and to teach him how to intelligently prescribe such drugs whenever he deems it advisable? For instance, if we consider it advisable to give a medicine for the treatment and cure of an alveolar abscess, or for the relief of a toothache, we should know the reason *why* we give it, and it only comes from a patient study of the action of the drug and the laws which govern it. That is why we cannot make use of any one drug as a specific for the treatment of toothache, and say, when prescribing it, "I know this medicine is going to cure this toothache, because some one else has used it and it proved effectual;" and why not? Because, very likely, in nine cases out of ten it will not be homœopathic to the case, and it is for this reason that there never can be any specific for toothache or for anything else. I do not mean to say, gentlemen, that I have always been successful in the giving of drugs in my practice, in all the cases in which I have felt called upon to prescribe, but I do often have patients and such conditions to treat where, without the aid of medicine, I simply cannot do anything for them. In such cases, following out Dr. Briggs's advice as given a year ago, we would advise the patient to go home and look well to his diet, and pay special attention to the laws of hygiene. That is all very well so far as it goes, but when a patient comes to you suffering with a violent toothache, and you are powerless to relieve it by mere mechanical or surgical methods, then is the time when you require a knowledge that is something more than mechanical, or you will be obliged to send the patient to a physician or somebody else who can relieve him.

It was only by having such cases in my practice that I was first led to take up a closer study of the materia medica, and I began by taking one drug at a time, studying its properties and noting carefully its effects. As most of you here know, I take the ground that the homœopathic way of prescribing is the only truly scientific one. Dr. Briggs was just saying to me while at dinner, "How do you explain the action of the drug? How do you *know* that such a power of the drug does the work?" That question does not interest me so much as does the fact of seeing the drug which is given produce relief that is almost or quite instantaneous; that is the thing which we are most interested in. It is not the question of the potency of the drug or its method of action. For instance, mercurius is a very valuable medicine, to be given not only for certain forms of abscess, but for certain kinds of toothache.

Now, in a toothache where mercurius is homœopathic to the case, no matter what potency is given, whether it be in the high or in the very low form, it will do good work. You may have another kind of toothache where mercurius will not have the slightest effect in relieving or stopping it, and why not? Simply because it is *not* homœopathic to the case. It is only by studying the properties and action of each drug and comparing them with the properties and action of other drugs, as for instance, comparing mercurius with bryonia or hepar, sulphur or aconite, or any other drug, which really enables us to apply a knowledge of materia medica intelligently and accurately. And it is only in so far as we are able to differentiate the properties and action of different drugs that they will be of any assistance to us where all other means and methods fail.

*Dr. Williams.*—I had an idea that some dependence was placed on the shaking or vibrating which increased the "potency" so called. Is not that a part of it? I would like to ask Dr. Taft if he can tell me if there is any influence of the vibratory action in producing the potency, and if so, if he can explain in what way it effects the drug?

*Dr. Taft.*—That, I am very sure, is a matter of experience among different homœopathic physicians. Very many of what are known as the Hahnemannians or strict homœopaths feel that it is better not to shake the solution even when the bottle in which it is contained gets nearly empty. For instance, when they are using a drug and the vial is nearly empty, they add the proper amount of sugar of milk to that bottle and find the power or force of the medicine which is still in the bottle is sufficient to permeate the whole, and the medicine has the same curative power as it would have if the solution were to be shaken up. But this is a matter of individual practice and experience among physicians. However, this is foreign to the subject of the paper, Mr. President, and I hope to hear the paper itself discussed.

*Dr. Briggs.*—I was sorry that I was called out as Dr. Taft was speaking, and therefore do not know just what to say in reply.

When I advocate dental students understanding thoroughly the principles that underlie the action of all medicines on the system in health and disease, it is to enable them to practise better their specialty and not to practise general medicine, and I have taken the point, as I did in that paper, that in all things that pertain strictly to his specialty he should know what to do and why he does it. But when it comes to the practice of general medicine, it seems to me that no specialist can undertake the general treatment of patients who require his services in a special direction; that the value of his knowledge is more in the timely reference of his patient to the physician than for the purpose of prescribing himself. My point is not by any means to make a medical practitioner of the dentist, but to insist that as a specialist he should understand the underlying principles of medicine.

*Dr. Taft.*—I simply take the ground, Mr. President, that a dentist should never go outside of what he feels is his legitimate field in the prescribing of any drug; and in the treatment of such cases as Dr. Bradley has referred to I think that many of them do come entirely within our province for treatment with the ordinary dental therapeutic agents at our command. I believe that in a great many instances where the dentist is an M.D., for example, and one who has a sufficient knowledge of how to diagnose cases, that such a man has a perfect right to administer remedies systemically to patients upon whom he is operating. But whenever cases arise where the dentist feels that it is unnecessary to understand well the constitutional condition of his patient before prescribing, he had better in all cases turn his patient over to the physician. I have often wished, while doing what I could for the patient from a dental stand-point, that I had the knowledge to prescribe such remedies as would cure certain conditions often found in the oral cavity, as pyorrhœa alveolaris, for instance, but I have never attempted to treat the patient systemically, for I believe that in such cases I would be going outside of our legitimate field.

*Dr. Williams.*—Speaking of being an "M.D.," one would think that by the affix of a certain title you have the possession of certain qualities, just as the stamp on a coin indicates its value. What difference would it make if a man had a knowledge of principles whether his title was M.D., D.M.D., or D.D.S.? Would not a thorough knowledge of principles as Dr. Taft has advocated be more important than the name by which that knowledge is called?

*Dr. Taft.*—Of what value is knowledge of any kind to a man if he cannot make a practical use of it? When we are taught any one thing in our specialty, it is with the expectation that we shall make some practical use of it. Of course, our patients in coming to us do not expect that we are going to take any chances or do anything which would place their lives or health in jeopardy, but when we feel that we are working and practising intelligently in the field of therapeutics, then I claim that we have not only a perfect right, but that it is our duty so to do.

*President Smith.*—If the members have nothing further to say, I will call upon Professor Greenleaf to close the discussion.

*Professor Greenleaf.*—Gentlemen, I wish to thank you for your attention. It was quite a question what kind of a paper to present to you in response to the invitation of your Executive Committee. This very subject could have been subdivided and a paper written on almost any of its special topics, but it seemed to me that it would be more useful to take it from this broader point of view, and I am very glad to see that you so earnestly approve of the inculcation of a sound knowledge of the principles underlying your specialty, although some may lead more directly in their application to other fields of thought. I will not review the discussion in detail, but I wish to say to Dr. Bradley regarding the question of counteracting the presence of uric acid or anything of that sort in the blood.

I intended to express the idea in my paper that a study of the principles underlying such a condition and its treatment was of value to the dentist not for purpose of treatment, but to aid him in recognizing the presence of such a disordered state even if not exactly diagnosing it, and in knowing whether or not it affected the existing dental disease, also as a guide as to whether it was best to refer the patient to a physician.

Regarding Dr. Bradley's other point,—viz., his not liking to have patients, whom he has referred to a physician, returned with the answer, "Nothing is the matter," when it is obvious to him that there is,—I can see that sometimes this way may prove a somewhat delicate question. as patients sometimes show so little care in the selection of their medical advisers. Unfortunately there "are empirics" and "make-believes" in medicine to-day and doubtless many patients are in their hands. Nevertheless, there are plenty of thoroughly-trained, able physicians whose answer as to "what is the matter" could be depended on. A safe rule for a patient or dentist to be guided by is to take the same pains in their selection that life insurance companies and medical schools take in selecting the physicians to answer their questions.

There is one other point that I wish to notice. It is too late and the subject is too extensive to discuss it with any detail, but I think it is fitting to make a very concise answer to certain of the questions brought forward by Dr. Taft, relating to the value of what he has called homœopathic therapeutics.

You have seen how a drug is studied by a scientific physician, and from his investigations we learn that large doses produce toxic effects; that the administration of certain doses—for instance, one-sixtieth of a grain of strychnia—produces certain effects which we call therapeutic; that a single dose of a smaller amount may produce an effect for a short time; but as the dose grows smaller no appreciable effects on the system are produced. These are not matters of guesswork; they are not matters of "belief;" they are matters of actual test and experiment as exact as anything that has been proved in the chemical laboratory. It would seem useless to pursue the discussion beyond this point, but it is entered into so honestly and earnestly at times that we sometimes feel it necessary to do so.

Much smaller doses of that very medicine, strychnia (under the name nux vomica, of which strychnia is an alkaloid), have been given with the "belief" that certain results have followed from them, and many believe, as Dr. Taft has said, that the more nearly infinitesimal the dose the better the results. One of the most prominent men in this so-called "school of medicine" has shown the fallacy, in his own words, of this belief in the high potencies, at least as regards triturations, after having pinned his faith on their great strength and prescribed them for years. He took some powered medicine, divided it into ten parcels, and to each one of those parcels added sugar of milk. He then subdivided each one of these into ten parts and added sugar of milk to them, and so on, until presently he had a degree of subdivision representing, as I call it, one-one-thousandth of the original strength; he now brought his microscope to bear on the powders, and with what result? In the first few packages he found traces of the drug, the next few parcels of the powder were found not to contain any traces of the drug whatever, and so on throughout his examination. Some parcels contained the drug and some did not, though he had hitherto been prescribing his powders considering that all had been very potent. He very honestly published his results, and from that time relinquished his faith in such "high potencies."

It is obvious that the "beliefs" of a character permitting such radical fallacies do not permit of serious scientific discussion, and while I might be willing privately to demonstrate their mistakes to any one honestly entertaining such views, it does not seem to me necessary or wise to publicly discuss them. Certainly it is not included in the scope of the paper which I have had the honor of presenting to you this evening.

WILLIAM H. POTTER, D.M.D.,
*Editor American Academy of Dental Science.*

